# Unraveling the spatial distribution and influencing factors of ‘Bengke’ traditional houses in Luhuo County, Western Sichuan

**DOI:** 10.1371/journal.pone.0314242

**Published:** 2024-12-16

**Authors:** Siwei Yu, Ding Fan, Ma Ge, Zihang Chen

**Affiliations:** 1 School of Art and Design, Le Shan Normal University, Leshan, Sichuan Province, China; 2 Institute of Chinese Studies, Hang Chiang University College, George Town, Penang, Malaysia; Macau University of Science and Technology, MACAO

## Abstract

The article examines the spatial distribution characteristics and influencing factors of traditional Tibetan “Bengke” residential architecture in Luhuo County, Ganzi Tibetan Autonomous Prefecture, Sichuan Province. The study utilizes spatial statistical methods, including Average Nearest Neighbor Analysis, Getis-Ord Gi*, and Kernel Density Estimation, to identify significant clustering patterns of Bengke architecture. Spatial autocorrelation was tested using Moran’s Index, with results indicating no significant spatial autocorrelation, suggesting that the distribution mechanisms are complex and influenced by multiple factors. Additionally, exploratory data analysis (EDA), the Analytic Hierarchy Process (AHP), and regression methods such as Lasso and Elastic Net were used to identify and validate key factors influencing the distribution of these buildings. The analysis reveals that road density, population density, economic development quality, and industrial structure are the most significant factors. The study also highlights that these factors vary in impact between high-density and low-density areas, depending on the regional environment. These findings offer a comprehensive understanding of the spatial patterns of Bengke architecture and provide valuable insights for the preservation and sustainable development of this cultural heritage.

## Introduction

Tibetan culture, a valuable heritage of the Chinese nation, is renowned worldwide for its unique architectural style, embodying profound historical and cultural significance. The traditional “Bengke” houses, unique to the Tibetan areas of Western Sichuan, not only reflect the intelligence and skilled craftsmanship of the Tibetan people but also demonstrate their remarkable adaptability to extreme natural environments [1, 2]. However, these traditional buildings face unprecedented challenges with the acceleration of globalization and modernization [3]. Systematically studying the spatial distribution characteristics and influencing factors of Bengke architecture is crucial for understanding how the natural environment and sociocultural dynamics shape the distribution of these dwellings [4, 5]. Such insights are essential for developing effective strategies to preserve and sustain this valuable cultural heritage [6, 7]. This study conducted various spatial statistical analyses, including average nearest neighbor analysis, hotspot analysis, and kernel density estimation, to investigate the clustering patterns and spatial autocorrelation of the “Bengke” buildings. Additionally, regression analyses, such as Lasso regression and Elastic Net regression, were utilized to thoroughly explore a series of environmental, social, and economic factors to identify and validate the key factors influencing the distribution of buildings. Through this multilevel analytical framework, the study aims to uncover the spatial characteristics of buildings and the complex mechanisms underlying these patterns.

## Literature review

Abundant research exists on the morphological structure and spatial patterns of traditional dwellings [8–10]. However, studies specifically focused on Tibetan traditional dwellings, particularly Bengke architecture, remain relatively scarce, especially concerning spatial distribution characteristics. Most existing research has concentrated on other forms of Tibetan architectural morphology [2, 11], spatial characteristics, and functional organization [12–14]. For instance, some scholars combined spatial analysis, comparative analysis, and field research to explore Tibetan prairie residences’ spatial characteristics and ecological adaptability [15], used space syntax to analyze the heterogeneity of Tibetan traditional dwellings in Gannan’s semi-agricultural, semi-pastoral areas, revealing the organizational modes of “sacred-secular” node spaces, structural differences in architecture, and the impact of regional culture on dwelling heterogeneity [16], and employed quantitative analysis and computer modeling to study the evolution of functional organization in Jiarong Tibetan traditional dwellings, showing how these dwellings have adapted to social and cultural changes over different historical periods [17]. These studies provide significant references for understanding Tibetan traditional dwellings’ spatial characteristics, functional organization, and cultural context.

Although these studies have made important advances in revealing architectural spatial structures and organization, their analyses often remain at a qualitative or single-dimensional level, failing to capture the complexity of architectural spatial distribution fully. Existing research typically lacks systematic analytical methods and comprehensive perspectives, especially in spatial statistical analysis [18]. Few studies have systematically used spatial statistical tools such as hot spot analysis, kernel density estimation, and Moran’s I to assess spatial distribution patterns and their autocorrelation [19–21]. Relying on a single analytical approach is insufficient for deeply understanding the research subject, as it limits the ability to identify distribution patterns of residential buildings and their potential interdependencies. Further research should introduce these systematic spatial statistical tools to uncover more complex spatial distribution patterns and the interactive relationships between factors [22, 23], providing a more comprehensive perspective on the spatial characteristics of residential buildings.

Moreover, Existing studies on the factors influencing the spatial characteristics of residential buildings have mostly focused on static analyses of single factors, with limited exploration of the complex interactions among multiple factors [24, 25]. For instance, some scholars primarily examined the impact of environmental factors on the structure and distribution of residential buildings in high-altitude areas, using regression models to analyze and validate these factors [17, 26]. Similarly, utilized GIS spatial graphics and quantitative analysis to investigate the organizational structure of Tibetan traditional village dwellings and the influence of cultural factors [3].

However, these studies often overlook the complex interplay among environmental, social, economic, and cultural dimensions, making it challenging to comprehensively capture the key factors influencing the spatial distribution of buildings. This limitation has resulted in insufficient depth in explaining the distribution patterns of architecture, highlighting the need for thorough validation of how multiple factors collectively influence building distribution characteristics [27, 28].

To address these limitations, this study will employ systematic spatial statistical tools and a multidimensional perspective to analyze the spatial distribution characteristics of Bengke architecture, aiming to identify the key factors influencing their distribution and interactions. By addressing gaps in existing research, this study seeks to provide a more scientific basis for the preservation and planning of these buildings.

## Methodology

### Research location

**Fig 1:**
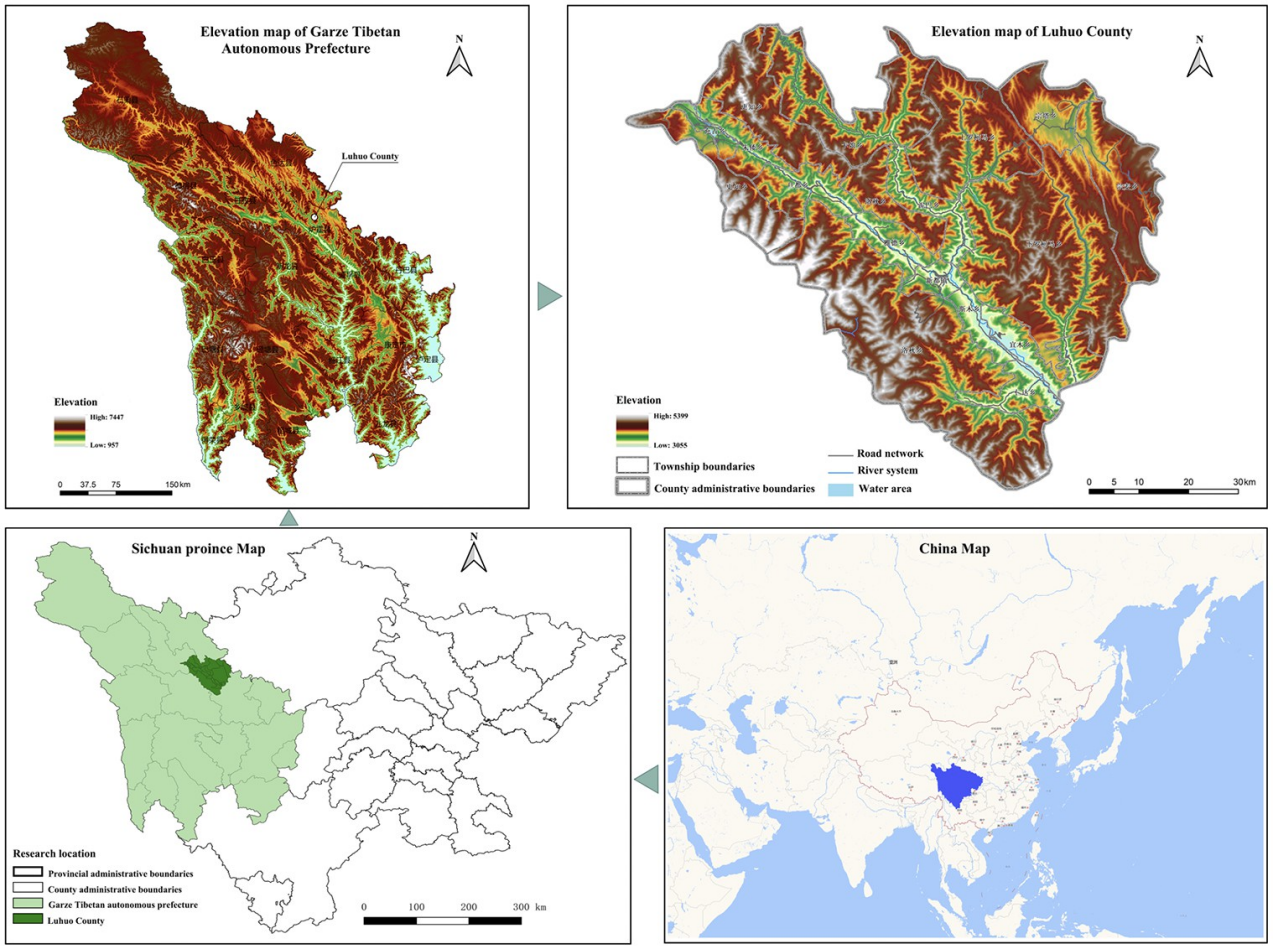
Research area (self-illustrated).

Luhuo County, located in the north-central part of Ganzi Tibetan Autonomous Prefecture on the southeastern edge of the Qinghai-Tibet Plateau, sits at an elevation of 3,860 meters, covering an area of 5,796.64 square kilometers. The county has 447,185 people across 18 townships (see Fig 1). This region is a significant center for Tibetan Buddhism and Kham Tibetan culture, contributing to its rich cultural landscape. Luhuo County is a major concentration area for “Bengke” residences, earning it the title “Capital of Bengke.” In 2021, in response to national policies such as the “Opinions on Further Strengthening the Protection of Intangible Cultural Heritage” and the “14th Five-Year Plan for Cultural Relics Protection and Technological Innovation,” Luhuo County actively established pilot villages for the protection of “Bengke” Tibetan residences, including Dawa Village and Xialatuo Village. These “Bengke” residences have been designated as provincial-level cultural relics and recognized as prefecture-level intangible cultural heritage.

### Research data and path

#### Data collection and processing

We selected 9 out of 18 townships in Luhuo County, Ganzi Tibetan Autonomous Prefecture, as our study sample through random sampling. Data collection took place from 22/04/2024 to 13/06/2024. Since this research primarily involved collecting publicly available spatial data, government reports, and observational data, it did not involve human participants or the recruitment of minors. Therefore, no participant consent was required. The study was reviewed and approved by the Academic Committee of the School of Arts and Design at Leshan Normal University, and it adhered to relevant research ethics guidelines and principles for conducting research involving publicly available data.

The data collected included a 30-meter resolution Digital Elevation Model (DEM), Points of Interest (POI) data (www.nbcharts.com/map/map.php), and coordinate information for Bengke residences (WGS-84). Additionally, information related to environmental, social, and economic activities in these townships was primarily sourced from Luhuo County government work reports (www.luhuo.gov.cn/zwgk), big data websites (www.gotohui.com/user/login.html), as well as literature and field surveys. By integrating quantitative data collection with qualitative observations, we ensured the comprehensiveness of the dataset.

The collected data were then preprocessed to ensure suitability for analysis. Non-digital data were encoded and missing or infinite values were cleaned to maintain dataset integrity. Subsequently, the data were normalized to eliminate the influence of differing data scales, ensuring that all variables contributed equally to the analysis.

#### Research path

This section outlines the methodological approach for analyzing the distribution characteristics of Bengke residences and their influencing factors. For the analysis of distribution characteristics, we primarily employed Geographic Information System (GIS) spatial analysis techniques, which included the following methods:

Firstly, the Average Nearest Neighbor Analysis method was used to determine whether the distribution pattern of the buildings is clustered, random, or uniform [29, 30]. The equation is as follows:

$$\begin{array}{c} R=\frac{Observed\;Mean\;Distance(d_{o})}{Expected\;Mean\;Distance(d_{e})} \end{array}$$ (1)

Here, $d_{o}=\frac{\sum_{i=1}^{n}d_{i}}{n}$, *d*_*i*_ represents the distance between the *i*th feature and its nearest neighbor, $d_{e}=\frac{1}{2\sqrt{\frac{n}{A}}}$, *n* is the number of features, and AAA is the area of the study region. The Average Nearest Neighbor (ANN) ratio *R* is used to interpret the distribution pattern: an *R* value less than 1 indicates clustering, greater than 1 indicates dispersion, and equal to 1 suggests a random distribution.

Next, the Getis-Ord Gi* hot spot analysis was employed to identify areas of high and low density in the distribution of traditional residential buildings [31, 32]. The equation is as follows:

$$\begin{array}{c} G_{i}^{*}=\frac{\sum_{j=1}^{n}w_{ij}x_{j}-\bar{X}\sum_{j=1}^{n}w_{ij}}{S\sqrt{\frac{n\sum_{j=1}^{n}w_{ij}^{2}-{\left(\sum_{j=1}^{n}w_{ij}\right)}^{2}}{n-1}}} \end{array}$$ (2)

Here, $G_{i}^{*}$ represents the Getis-Ord Gi* statistic for the *i*th feature; *x*_*j*_ is the attribute value of the jth feature; *w*_*ij*_ is the spatial weight between features *i* and *j*; ${\bar{X}}^{-}$ is the mean of all attribute values, calculated as $\bar{X}=\frac{\sum_{j=1}^{n}x_{j}}{n}$, and *S* is the standard deviation of the attribute values, calculated as $S=\sqrt{\frac{\sum_{j=1}^{n}{\left(x_{j}-\bar{X}\right)}^{2}}{n-1}}$.

Subsequently, Kernel Density Estimation (KDE) is used to calculate and visually display the density of spatial data [33, 34]. The equation is as follows:

$$\begin{array}{c} f\left(x,y\right)=\frac{1}{nh^{2}}\sum_{i=1}^{n}K(\frac{d(\left(x,y\right),(x_{i},y_{i}))}{h}) \end{array}$$ (3)

Here, (*x*, *y*) represents the location where the density is being estimated, *n* is the number of feature points, *h* is the bandwidth or smoothing parameter, *d*((*x*, *y*), (*x*_*i*_, *y*_*i*_)) is the distance between the estimation point and the *i*th feature, and KKK is the kernel function.

At the same time, Moran’s I is employed to preliminarily assess spatial autocorrelation, determining whether the distribution of residences exhibits spatial dependency [31, 35]. The equation is as follows:

$$\begin{array}{c} I=\frac{n}{\sum_{i=1}^{n}\;\sum_{j=1}^{n}\;w_{ij}(x_{i}-\bar{x})(x_{j}-\bar{x})} \end{array}$$ (4)

Where, *x*_*i*_ and *x*_*j*_ are the attribute values of the *i*th and *j*th features, *w*_*ij*_ represents the spatial weight between features *i* and *j*, and *n* is the total number of features. If the value of I is close to 1, it indicates positive spatial autocorrelation, meaning similar values are clustered together. If the value is close to −1, it indicates negative spatial autocorrelation, meaning similar values are dispersed.

These spatial statistical analyses provide strong support for understanding the spatial distribution patterns of Bengke residences. During the research process, technologies such as ArcGIS 10.8 and Python were employed in combination [36].

For the analysis of influencing factors, we used Exploratory Data Analysis (EDA), the Analytic Hierarchy Process (AHP), and Lasso and Elastic Net regression methods to extract and validate the key factors affecting the distribution of Bengke residences.

First, the dataset underwent coding and normalization to ensure consistency across the data. Next, EDA was conducted to perform an initial analysis, identifying the basic characteristics of the data. Descriptive statistics and visualization tools were used to understand data distributions and detect outliers [37, 38].

Subsequently, the Analytic Hierarchy Process (AHP) was applied to determine the weights of the influencing factors. A judgment matrix was constructed to quantify the relative importance between different factors, assigning a scale value from 0 to 1 to each pairwise relationship to reflect their importance relative to one another. Then, to calculate the weight vector *W* = (*w*_1_, *w*_2_, *w*_3_, …*w*_*n*_) in the Analytic Hierarchy Process (AHP), as follows:

$$\begin{array}{c} \text{AW}=\lambda_{\text{max}}\;W \end{array}$$ (5)

To ensure the reasonableness of the pairwise comparison matrix A in the Analytic Hierarchy Process (AHP), a consistency check is performed. This involves calculating the Consistency Index (CI) and Consistency Ratio (CR). The process is as follows:

$$\begin{array}{c} CI=\frac{\lambda_{\text{max}}-n}{n-1} \end{array}$$ (6)

$$\begin{array}{c} CR=\frac{CI}{RI} \end{array}$$ (7)

The consistency of the matrix is acceptable only if the *CR* value is less than 0.1. If the *CR* value is greater than 0.1, it indicates that the judgments are inconsistent, and the pairwise comparisons may need to be revisited and adjusted. This consistency check is crucial to ensure that the judgments made during the evaluation of the relative importance of different factors are robust and reliable [39, 40].

Finally, we proceeded with the extraction of influencing factors. Initially, we employed a weighted regression approach for the calculations [41, 42]. However, during preliminary estimates, we found that the model exhibited overfitting, as indicated by a high *R* value, leading to insufficient degrees of freedom. The limited sample size resulted in some statistical measures, such as the F-statistic and P-values, being uncalculable, with results showing as NaN. Given the small sample size, we attempted to extract the independent variables with relatively higher weights to construct a more streamlined model suitable for the dataset, but the results remained suboptimal.

To address the challenges posed by the small dataset, we adopted the Lasso regression method. This approach introduces regularization, which effectively resolves multicollinearity issues [43, 44]. Lasso regression simplifies the model, prevents overfitting, and enhances the model’s predictability and interpretability by optimizing the objective function [45]. Then equation as follows:

$$\begin{array}{c} \text{Min}L\left(\beta \right)=\sum_{i=1}^{n}{\left(y_{i}-\beta_{0}-\sum_{j=1}^{p}\beta_{j}x_{ij}\right)}^{2}+\lambda \sum_{j=1}^{p}|\beta_{j}| \end{array}$$ (8)

Here, *L*(*β*) represents the objective function, where *n* is the number of samples, *p* is the number of independent variables, *y*_*i*_ denotes the actual value of the dependent variable for *i*th observation, *β*_0_ is the intercept term, *β*_*j*_ refers to the coefficient of the *j*th independent variable, *x*_*ij*_ is the value of the *j*th independent variable for *i*th observation, *p* indicates the total number of independent variables, and λ is the regularization parameter that controls the intensity of the L1 penalty term [46].

To further confirm the importance of the variables, Elastic Net regression was employed for validation. This method combines the advantages of both L1 (Lasso) and L2 (Ridge) regularization, providing a more robust verification and optimization of the variable selection process [47]. Elastic Net helps refine and extract the most important key factors. The formula for Elastic Net is as follows:

$$\begin{array}{c} \text{Min}L\left(\beta \right)=\sum_{i=1}^{n}{\left(y_{i}-\beta_{0}-\sum_{j=1}^{n}\beta_{j}x_{ij}\right)}^{2}+\lambda (a\sum_{j=1}^{n}|\beta_{j}|+\frac{1-a}{2}\sum_{j=1}^{n}\beta_{j}^{2}) \end{array}$$ (9)

Here, $\sum_{i=1}^{n}{\left(y_{i}-\beta_{0}-\sum_{j=1}^{n}\beta_{j}x_{ij}\right)}^{2}$ represents the gap or difference between the model’s predicted values and the actual values. λ is the regularization strength parameter, which controls the intensity of the L1 penalty. *α* is the weight parameter that determines the balance between L1 regularization (the Lasso component) and L2 regularization (the Ridge component). When *α* = 1, Elastic Net regression simplifies to Lasso regression, focusing solely on the L1 penalty. $\sum_{j=1}^{n}|\beta_{j}|$ represents the L1 regularization component, $\frac{1-a}{2}\sum_{j=1}^{p}\beta_{j}^{2}$ is the L2 regularization component, which prevents the model coefficients from becoming too large by penalizing their magnitude [48].

To evaluate the predictive ability of the model, we used k-fold cross-validation in Python. This method divides the entire dataset into K subsets, with each subset maintaining the statistical properties of the data as much as possible. In each iteration (denoted as the ith iteration, where *i* = 1, 2, …, *k*), one subset $D_{{\text{valid}}_{i}}$ is chosen as the validation set, while the remaining *K* − 1 subsets $D_{{\text{train}}_{i}}$ are used as the training set. The model *M*_*i*_ is trained on the training set and then evaluated on the validation set $D_{{\text{train}}_{i}}$ to obtain the performance metric *P*_*i*_. The overall performance of the model is assessed by calculating the average of the performance metrics across all iterations, denoted as *P*_*avg*_ [49]. The equation as follows:

$$\begin{array}{c} P_{avg}=\frac{1}{k}\sum_{i=1}^{k}P_{i} \end{array}$$ (10)

Here, *P*_*i*_ represents the performance metric for the ith iteration, such as Mean Squared Error (MSE). The value of K is typically set to 5 or 10 to ensure the stability of the cross-validation process and the reliability of the model evaluation [44].

This research approach, from a comprehensive methodology and multi-factor analysis perspective [50], allows for a thorough identification of the spatial characteristics of Bengke residential distributions while also quantifying the influence of various factors on distribution patterns. By utilizing a multi-level analytical framework, this study effectively overcomes the limitations of single-method approaches, ensuring the robustness and accuracy of the analysis results. Ultimately, this approach provides a reliable quantitative basis for uncovering the key factors influencing the spatial distribution of traditional residences [51], laying a solid foundation for further research and practical applications in related fields.

## Analysis and result

### The characteristic of *Bengko* buildings

**Fig 2:**
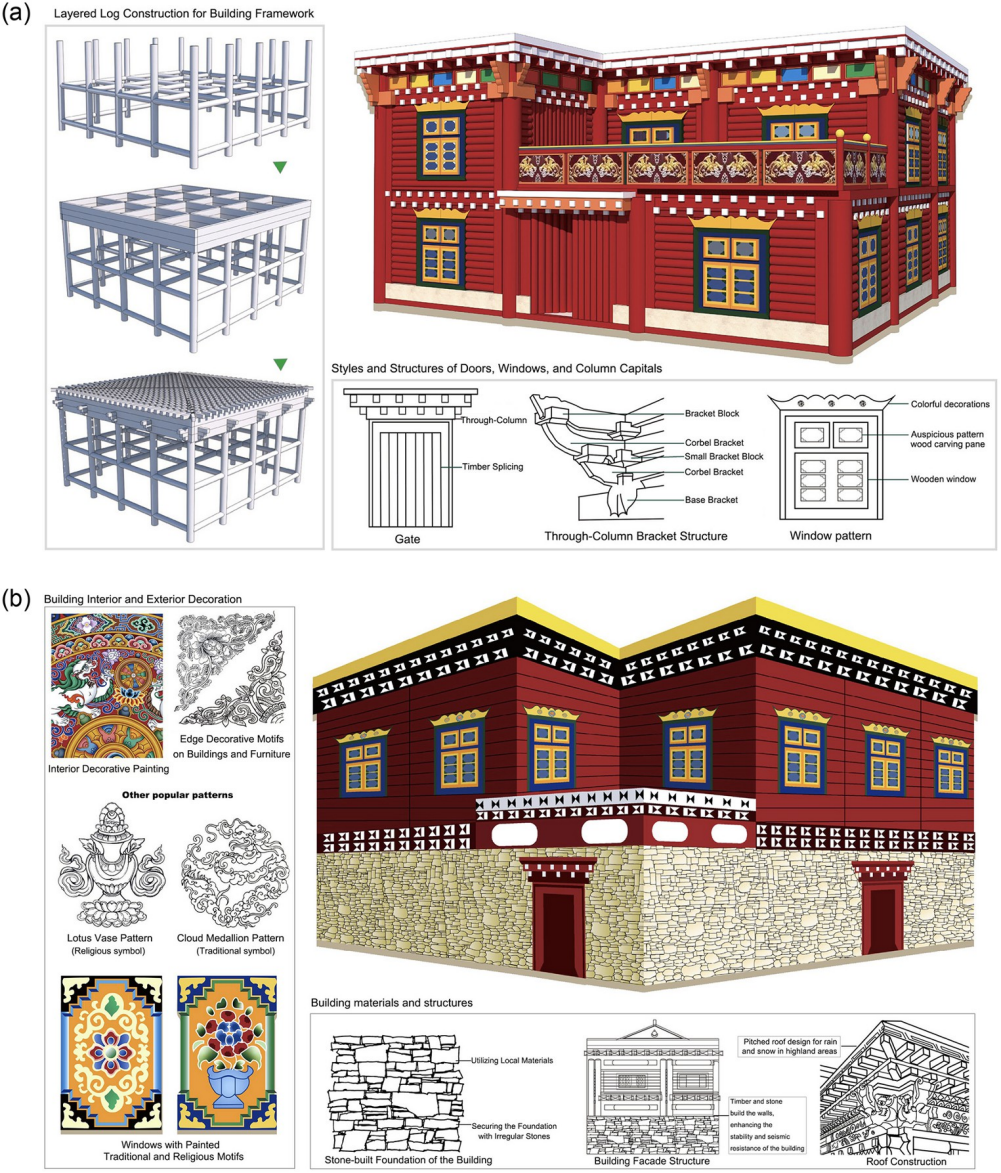
Bengke architecture (self-illustration).

Tibetan residences exhibit diverse structural forms due to regional and tribal differences, such as the “Danba Tibetan Villages” of the Jiarong Tibetans, the “Muya Tibetan Houses” in Xinduqiao, and the “Flat-roofed Fortified Houses” of the Kham Tibetans. Among these diverse architectural types, the “Bengke” buildings in Luhuo County, Ganzi Tibetan Autonomous Prefecture, Sichuan Province, stand out as typical stilted-style traditional Tibetan dwellings (as seen in Fig 2), widely distributed across the county’s various townships.

“Bengke” is a Tibetan word meaning ‘stacked wood’. The construction of the houses takes full advantage of the rich forestry resources in the area and adopts the traditional Chinese mortise and tenon technique. Some are constructed of stone and wood, while others are made entirely of logs (as shown in Fig 2a and 2b). These constructions demonstrate excellent carpentry skills and give the buildings remarkable resistance to earthquakes and durability. The area of a Bengke building is uniquely calculated using “kong” as the unit, where the space enclosed by four column tops is considered one “kong,” approximately 10 square meters. Typically, a building with four columns covers about 90 square meters, while one with six columns covers about 120 square meters. These buildings are usually two-story structures. The ground floor, originally used for storing goods or housing livestock, is now often converted into a living room or kitchen due to social and economic development. The second floor serves as the main living space, typically arranged around a central courtyard or in a “U” shape, with functional rooms such as bedrooms and a chapel. This design meets the practical needs of Tibetan families and reflects their emphasis on life and religion. Externally, Benguet buildings are dominated by a dark red color, and window and door frames are often decorated with colorful paintings that contrast with the natural surroundings [52]. According to local historical records, Bengke residences in Luhuo County have a history of over a century.

However, with the implementation of the policy of prohibiting the cutting down of natural forests, the construction of Bengke buildings has been gradually restricted, and the culture of the region in which they are embodied is in danger of disappearing. Therefore, this study examines Bengke architecture from the perspective of spatial distribution characteristics, exploring its connections with the natural environment and sociocultural activities. This approach not only provides a scientific perspective for the preservation of this valuable intangible cultural heritage but also lays the foundation for further investigation into its importance in cultural transmission within Tibetan regions.

### Spatial distribution characteristics of “Bengke”

**Fig 3:**
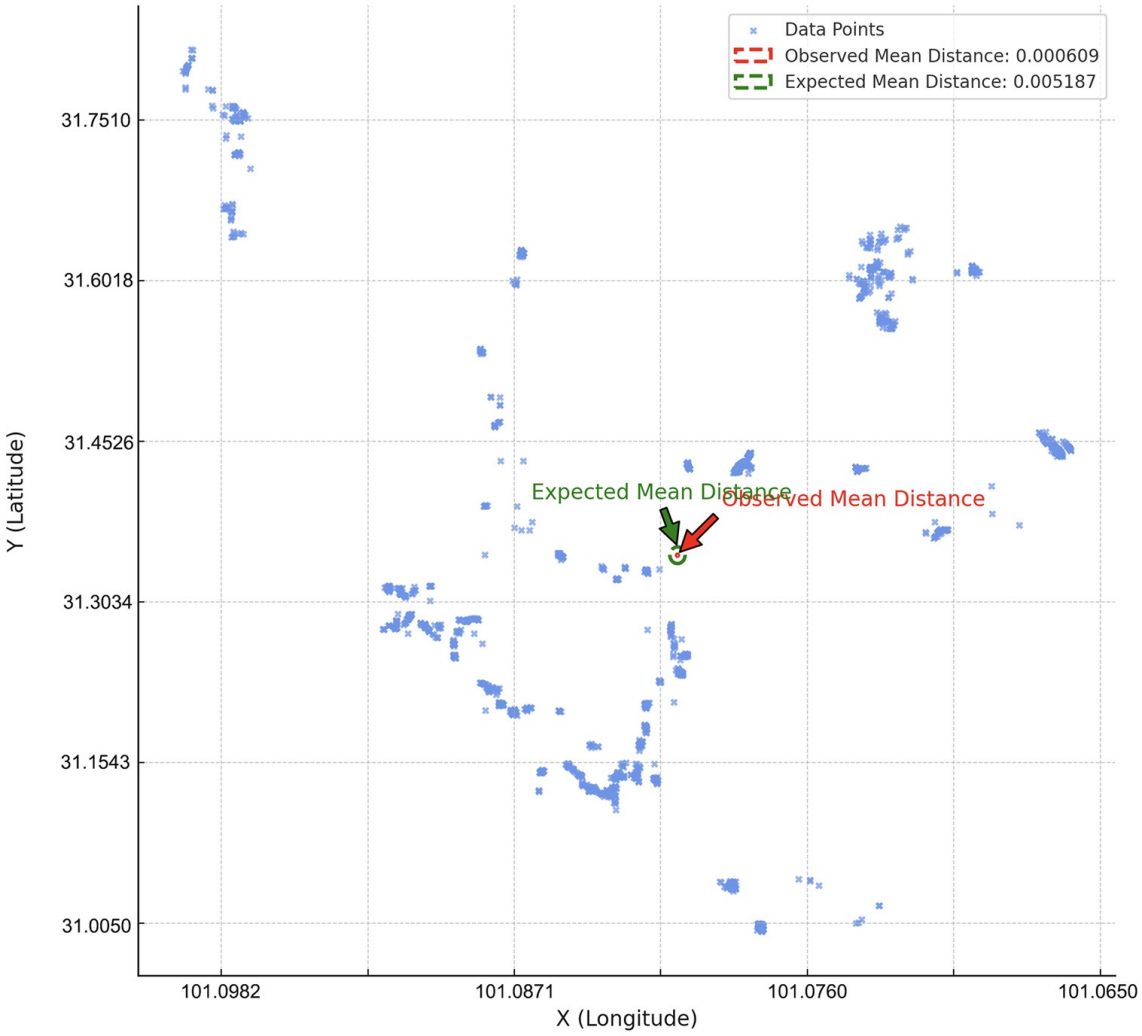
Scatter plot with observed and expected mean distance circles(self-illustration).

This section provides a detailed analysis of the spatial distribution characteristics of Bengke buildings using spatial statistical methods. Based on field survey data recording the locations and numbers of Bengke buildings, we used BIGMAP and collected the coordinates of 1,825 buildings in 10 major clustering townships of Bengke dwellings, including Xialatuo, Kaniang, Yade, etc., in Luhuo County. Through Average nearest-neighbor analysis, we discovered significant clustering in the spatial distribution of these buildings (as seen in Fig 3). Specifically, the observed mean nearest neighbor distance is significantly shorter than the expected distance for a random distribution [30], indicating clear clustering tendencies.

This finding suggests that Bengke buildings are typically located close to each other, with site selection likely influenced by factors such as elevation gradient, environmental resources, and socioeconomic activities rather than being randomly distributed. The dense distribution in specific areas may reflect a strategic adaptation to local environmental conditions and the practical needs of production and daily life.

**Fig 4:**
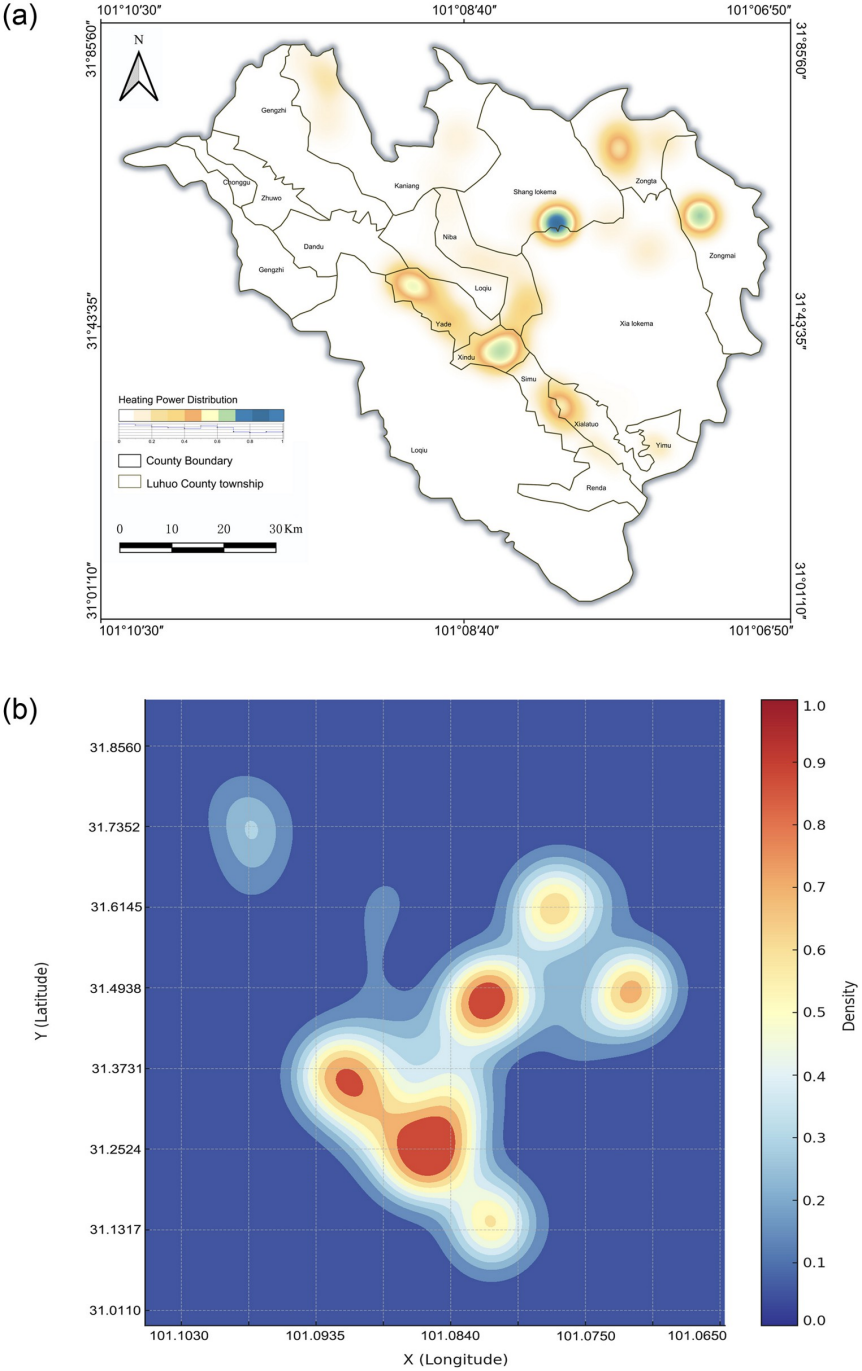
Spatial statistical analysis(self-illustration).

To further verify the findings, we employed hot spot analysis and kernel density estimation methods (see Fig 4a and 4b). The hot spot analysis revealed that Bengke buildings form significant high-density hot spots in areas such as Shangluokema Township and Xindu Town, while cold spots appear in more remote regions. This distribution pattern is likely closely related to variations in resource distribution, infrastructure development, and socioeconomic activities within the region [53]. The kernel density analysis further confirmed these observations [33], showing that the high-density areas align with the results of previous analyses, thereby validating the spatial clustering and dispersion of Bengke buildings in specific regions.

Building on this, we proposed a new hypothesis: whether the distribution of these buildings may exhibit spatial autocorrelation. To explore this question further, we collected land area data for each building within the main clusters and used Moran’s I to test for spatial autocorrelation in building density. This analysis aimed to assess whether buildings with similar densities are spatially clustered or dispersed and whether there is a significant clustering phenomenon in their spatial structure [36, 54].

**Fig 5:**
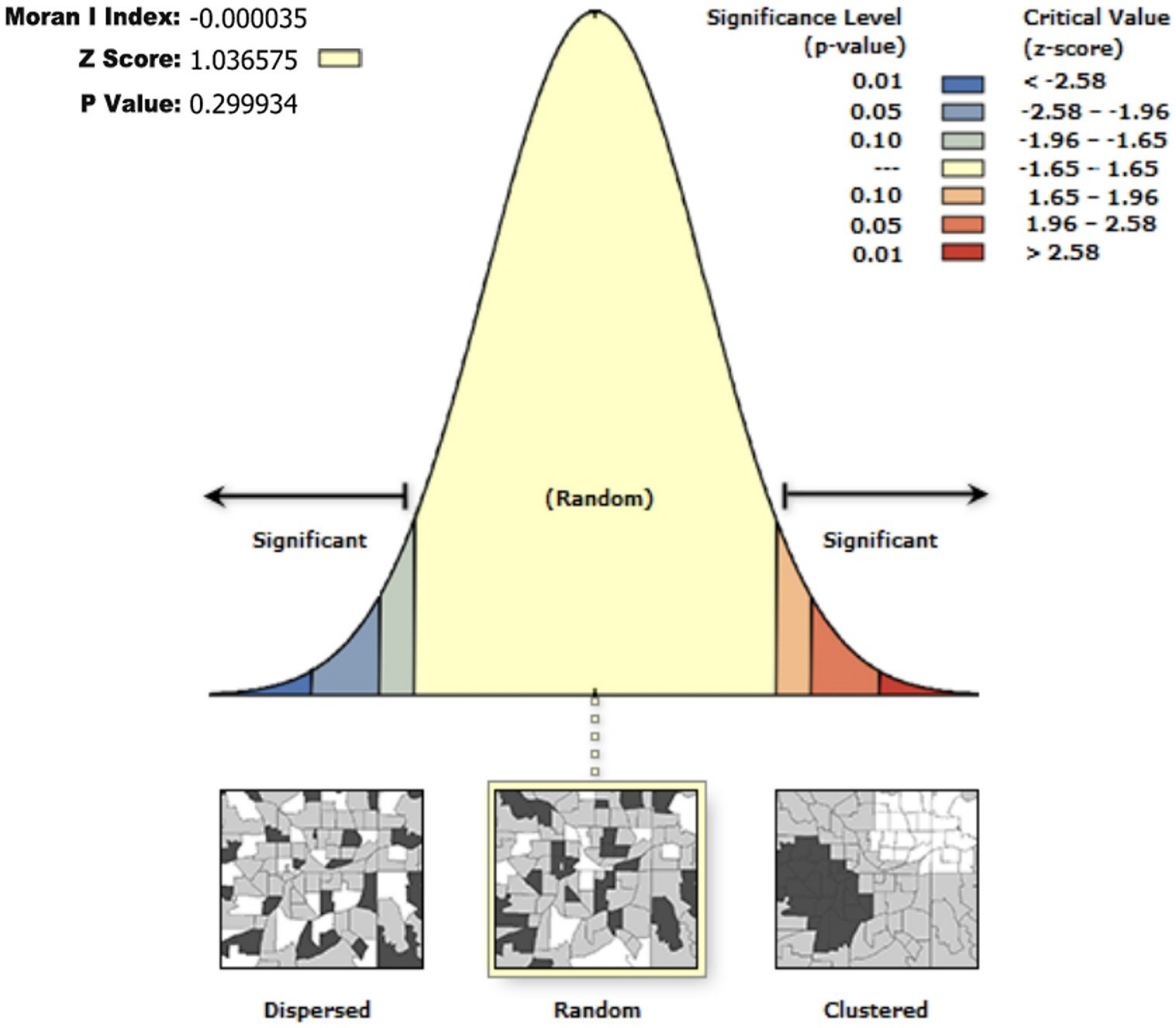
Spatial autocorrelation based on Moran’s index(self-illustration).

However, the calculation results (as shown in Fig 5) indicate that Moran’s I index is close to zero, suggesting that the spatial distribution of Bengke buildings does not exhibit significant autocorrelation. The Z-score is near the center of the normal distribution, further supporting the randomness of the spatial distribution. Additionally, the P-value is well above 0.05, indicating statistical insignificance. Therefore, we can preliminarily conclude that the buildings do not show spatial autocorrelation, implying that the factors influencing their distribution are likely more complex and not simply manifested in geographic spatial clustering or dispersal patterns.

### Extraction of impact factors

In this section, we conduct an in-depth analysis of the key factors influencing the spatial distribution of Bengke buildings. We begin with an exploratory analysis to preliminarily identify potential driving factors. Next, we determine the importance of each factor through weight calculation. Building on this foundation, we apply a combination of methods, including Lasso regression, Elastic Net regression, and cross-validation, to extract the variables that significantly impact the distribution of these buildings from a range of candidate variables. The comprehensive application of these methods will help uncover the complex mechanisms influencing the distribution of Bengke buildings.

**Fig 6:**
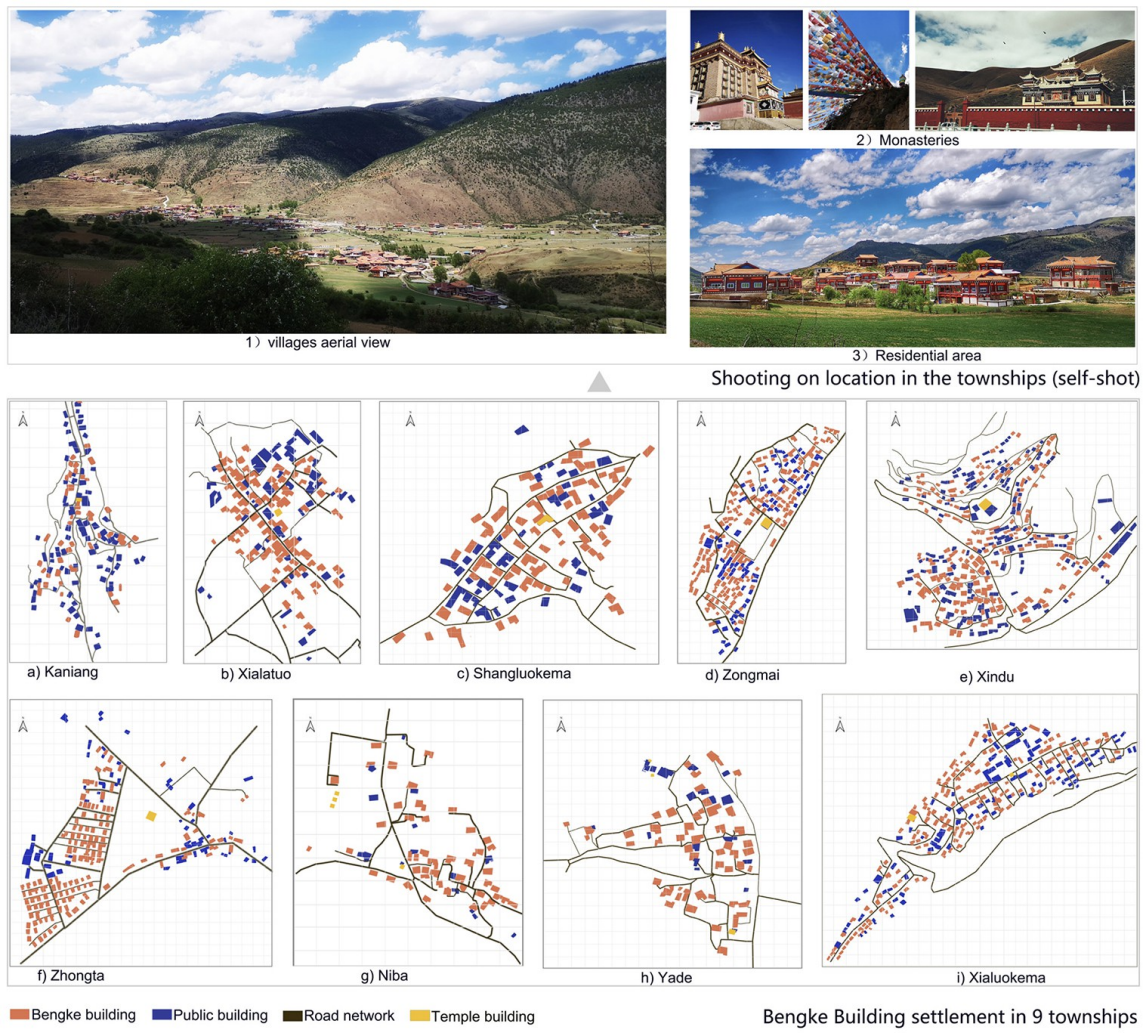
Geospatial map of buildings settlements in localized areas of 9 townships.

Before delving into the specific relationships between the variables, Fig 6 provides an overview of the spatial distribution of Bengke buildings in the nine surveyed townships. The figure illustrates the distribution of Bengke dwellings about public buildings, road networks, and temple buildings, providing a detailed geographic perspective of these architectural clusters. By visualizing the location of these buildings about the surrounding infrastructure and religious sites, the map helps to provide contextual information for the spatial patterns that will be analyzed in detail by subsequent statistical methods. It is worth noting that the spatial clustering trends that can be discerned from the map, especially in areas of high road density and proximity to temples, may be suggestive of the underlying characteristics of the building clusters.

As shown in Fig 6, Bengko buildings tend to form dense clusters, and the concentration of the road network and proximity to temple buildings appear to be influencing the spatial layout. The road network and public buildings depicted in the figure indicate the interaction between infrastructure development and public buildings, such as temples, and the traditional distribution of dwellings. These spatial relationships suggest that infrastructure (e.g., road density) and social-cultural elements (e.g., proximity to temples) may be important factors influencing the distribution of Bengke buildings.

**Fig 7:**
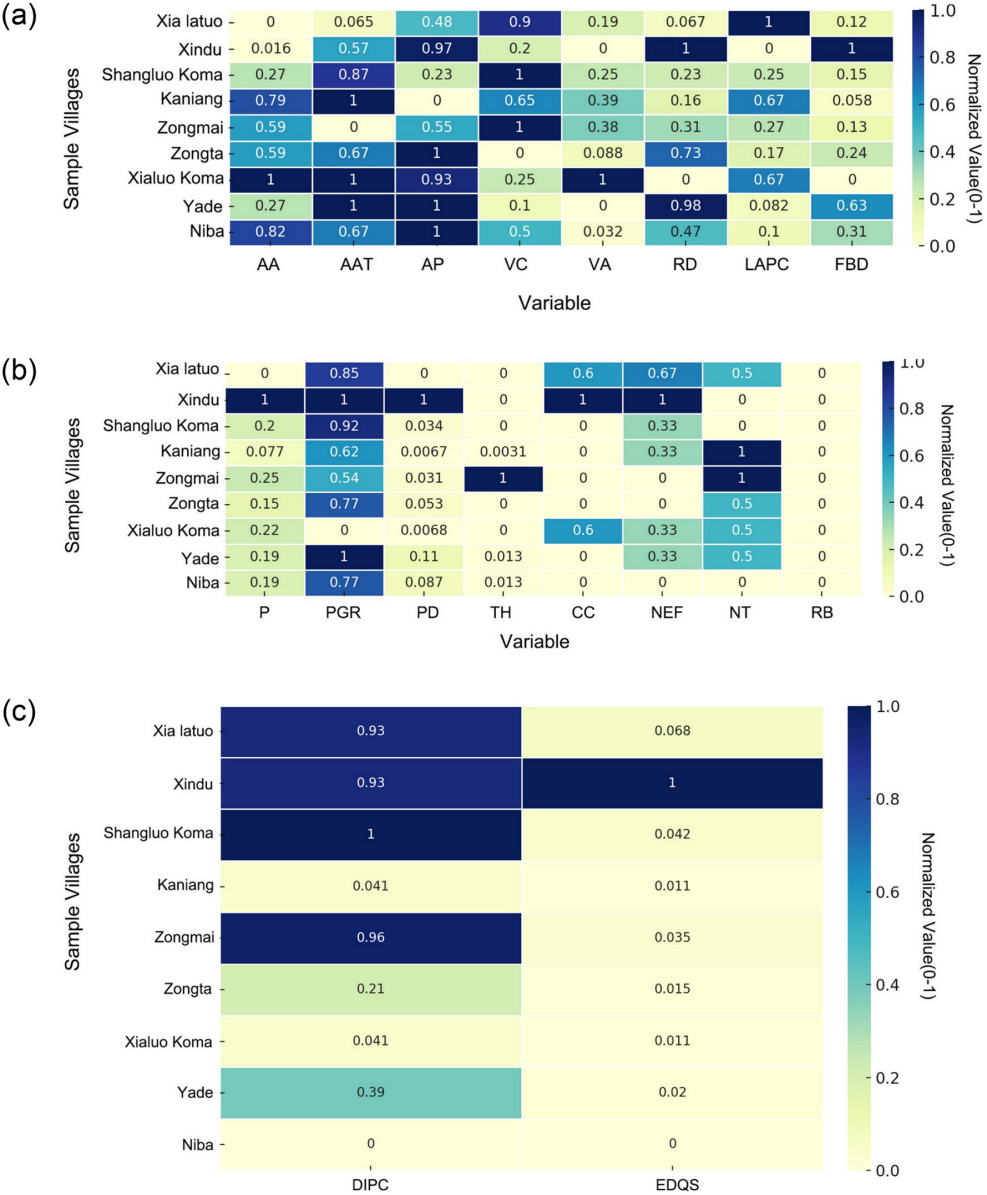
Distribution and comparison of environmental, social, and economic factors.

We collected environmental, social, and economic data from the sample villages and visualized their distribution and heterogeneity using heat maps. Fig 7(a)–7(c) illustrates the spatial distribution of these elements, where color intensity reflects the variable values across villages. Darker shades indicate higher values, while lighter shades represent lower values. This visualization highlights the disparities among villages in terms of natural conditions, social infrastructure, and economic development. For example, Fig 7a shows that villages at higher elevations tend to have lower mean annual temperatures and reduced vegetation coverage. In Fig 7b, the heat map displays villages with higher communication coverage and population density, while Fig 7c depicts variations in economic factors such as per capita disposable income and the quality of economic development. Using this spatial framework, we then apply statistical methods to quantify the relationships between these variables and the observed spatial patterns.

**Fig 8:**
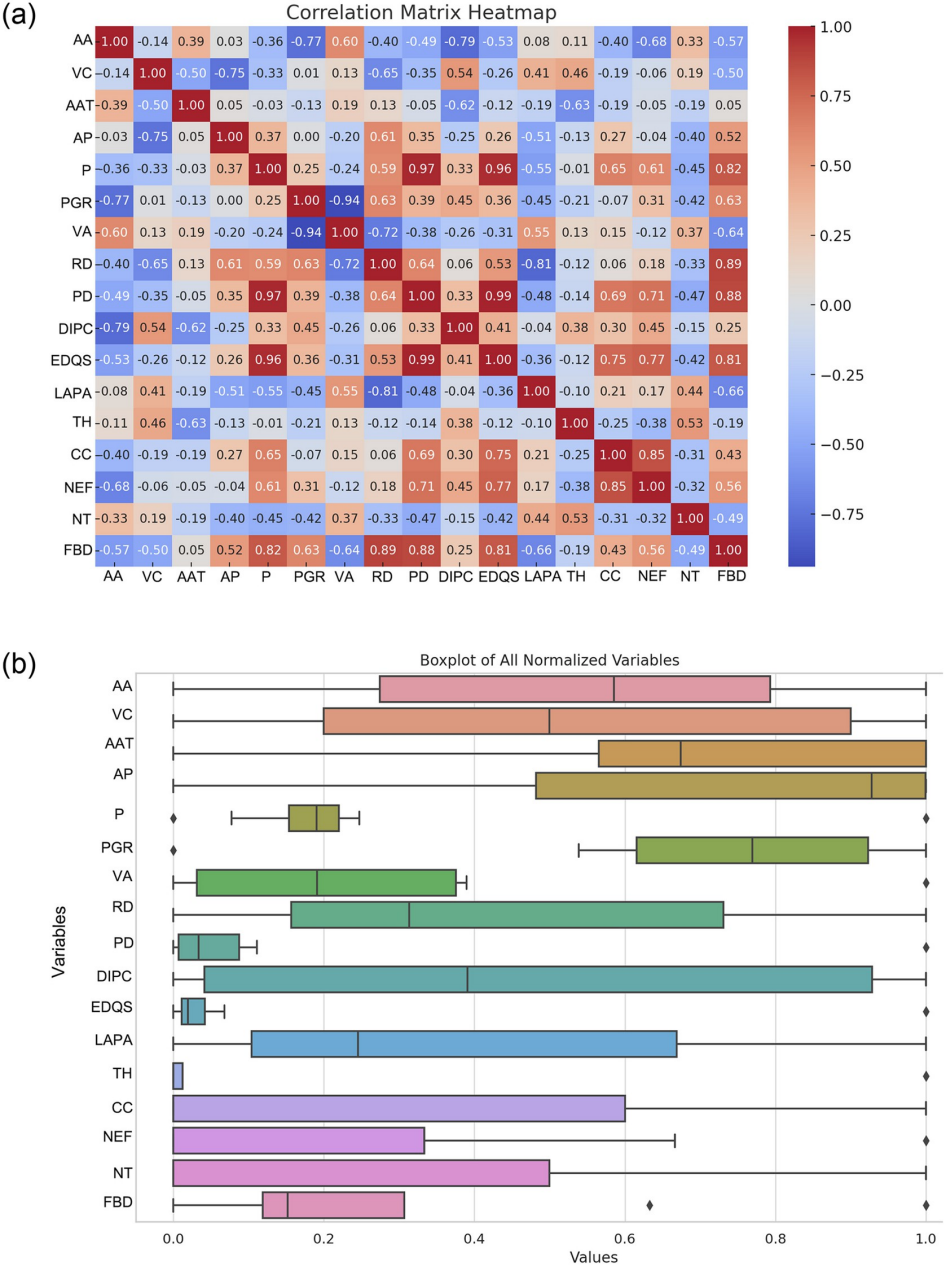
Exploratory analysis of variables (self-illustration).

Building on this spatial framework, we applied statistical methods to further quantify these relationships. In the following analyses, we examine how key variables—such as roadway density, population density, and economic development—correlate with the observed spatial patterns. The correlation heatmap in Fig 8a reveals linear relationships among several key variables [55]. For instance, population density, village area, and economic development quality score exhibit a high positive correlation, suggesting that these factors collectively drive regional development. Conversely, elevation shows a negative correlation with economic development levels, indicating that high-altitude areas may face challenges in economic growth. Additionally, the population growth rate is negatively correlated with natural factors such as average annual temperature and vegetation coverage, suggesting that these environmental conditions may inhibit population growth.

Traditional cultural factors (e.g., township history) show low or negative correlations with modern amenities (e.g., communication coverage, number of educational facilities), reflecting potential independence or conflict between tradition and modernization. Overall, the heatmap highlights the key factors influencing regional economic and social structures and their complex interactions. Notably, “folk building density” shows a significant positive correlation with variables such as “village area,” “road density,” and “economic development quality score,” indicating that these factors may be positively correlated with the density of Bengke buildings.

However, some variables like “relevant policies and regulations,” “religious belief,” “building age,” and “infrastructure level” show low correlations with other variables, resulting in no color coding on the heatmap. This may be due to the small sample size in the dataset or the limited range of variation in certain variable values. Nevertheless, this does not imply that these variables are unimportant in practice; rather, their potential impacts may require analysis through more complex nonlinear models [56].

The box plot in Fig 8b illustrates the standardized distribution of each variable, revealing regional differences and concentration trends [57]. For instance, the distributions of village area and disposable income are more dispersed, indicating significant regional disparities, whereas variables like population growth rate and communication coverage are more concentrated, showing less variability. Additionally, some variables, such as village area and per capita village area, have significant outliers, indicating particularities in certain regions. These outliers may require special handling in model analysis to avoid disrupting model fitting. Notably, some variables, such as “population density” and “economic development quality score,” exhibit significant distribution differences across regions, suggesting that their impact on “folk building density” may vary between regions. This conclusion will be further evaluated in subsequent analyses.

After conducting exploratory analysis, we have preliminarily identified several key variables that may have significant impacts (see Table 1). To further clarify the core correlation of these variables, the next step involves calculating their weight values using the Analytic Hierarchy Process (AHP). This process allows for the systematic evaluation of each variable’s importance in a multi-criteria decision-making environment, providing a more scientific basis for subsequent analysis and decision-making [58, 59].

**Table 1:** Weights of variables in the indicator system.

Layer	Variables	Weight
**Environment Factors**	Average altitude (m)	0.0356
	Vegetation coverage (%)	0.0271
	Average annual temperature (°C)	0.0158
	Annual precipitation(mm)	0.0257
	Village area(km^2^)	0.0323
	Land area per capita (km^2^/person)	0.033
	Road density (km/km^2^)	0.04
	Folk building Density	0.0252
**Social Factors**	**Population**	0.0426
	Population density (persons/km^2^)	0.0460
	Population growth rate (%)	0.0329
	Township History	0.0179
	Infrastructure Level	0.0175
	Communication coverage (%)	0.032
	Number of education facilities	0.035
	Level of social service facilities	0.0885
	Building age	0.0323
	Number of temples	0.0274
	Religious belief	0.0274
	Policies related to the preservation of architectural cultural heritage	0.1134
**Economic Factors**	Disposable income per capita	0.0277
	Economic Development Quality Score	0.0443
	Industrial structure	0.0175

(***Note:*** Consistency index (CI) and consistency ratio (CR) < 0.1)

**Fig 9:**
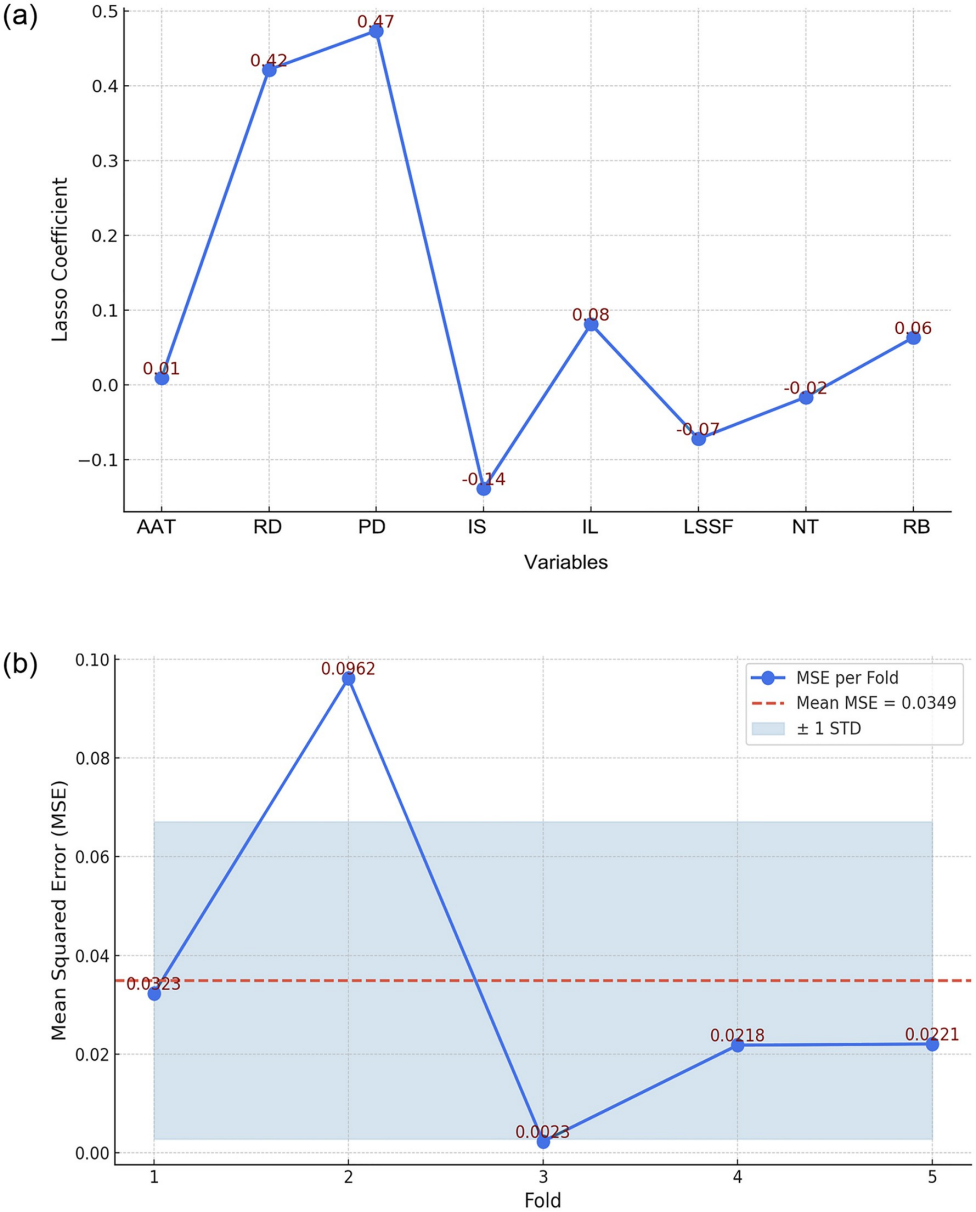
Estimation of regression coefficients for key variables and validation (self-illustration).

Next, we applied Lasso regression to filter out the variables with significant impact and employed cross-validation to ensure the model’s stability and predictive power (see Fig 9).

The results (see Fig 9) show that road density and population density are the most significant factors influencing the density of Bengke buildings, with larger coefficients and positive effects. This indicates that infrastructure and population distribution play a crucial role in driving the spatial clustering of Bengke buildings. Conversely, factors such as industrial structure and the level of social service facilities may have negative impacts on building density, suggesting that these variables might conflict with or inhibit traditional architecture during the modernization process.

To further validate the reliability of these results, we used the cvpartition function in MATLAB 2022b to divide the data, setting up a 5-fold cross-validation. The crosstalk function was employed to perform cross-validation and training [59]. We observed some variation in Mean Squared Error (MSE) across the folds, with the lowest MSE in the third fold (0.0023) and the highest in the second fold (0.0962). The overall average MSE was 0.0349, with a standard deviation (STD) of 0.0321, indicating low error and good fit, suggesting that the model exhibits strong stability.

**Fig 10:**
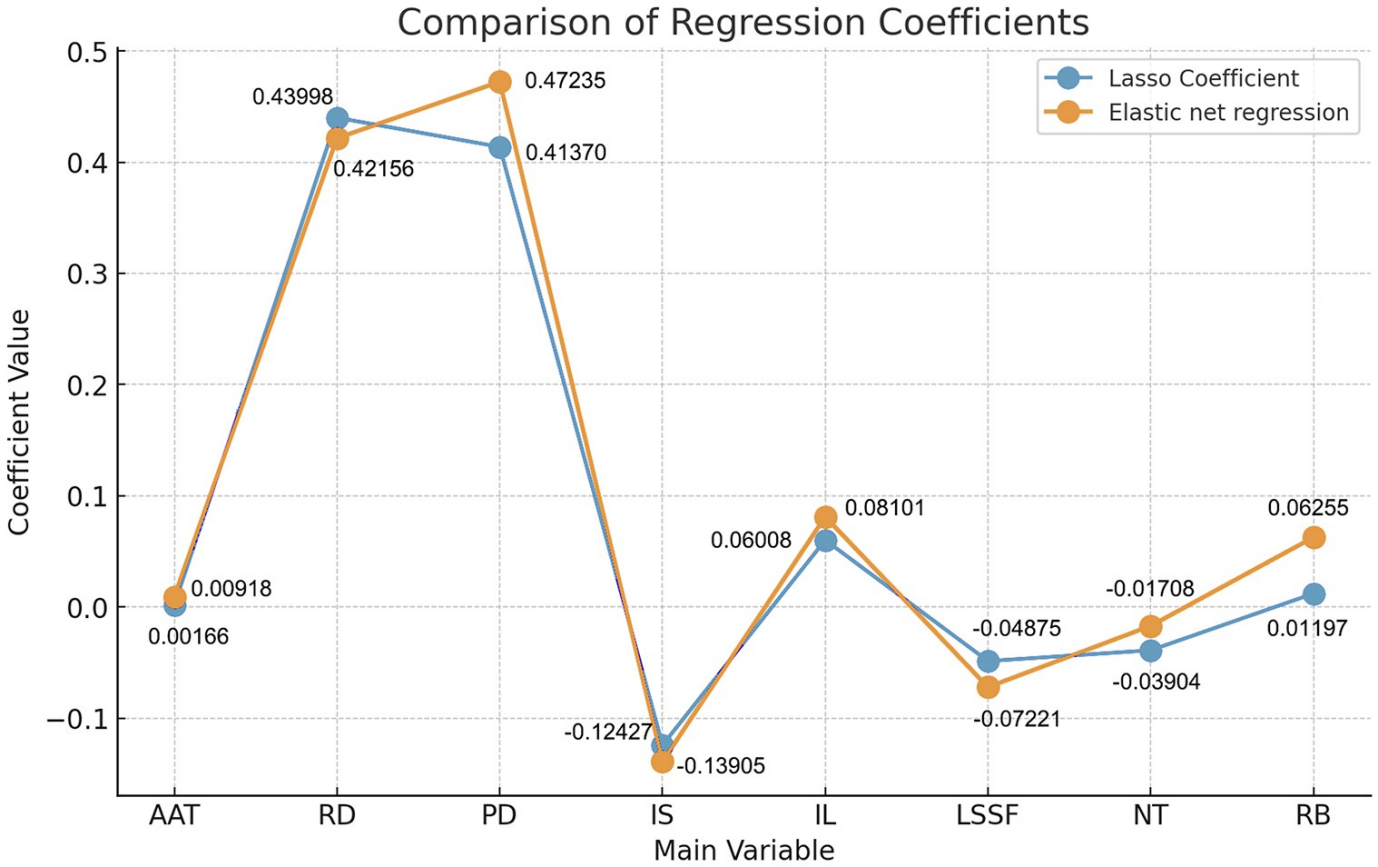
Comparison of Lasso and Elastic Net regression coefficients (self-illustration).

To further enhance the model’s accuracy, we applied Elastic Net regression for a more refined analysis. Comparing the coefficients from Lasso regression with those from Elastic Net, we found that Elastic Net provided more balanced and stable regression coefficients for each variable. This approach improved the model’s interpretability and predictive accuracy [45]. As seen in Fig 10:

Based on the combined analysis results from Lasso and Elastic Net regressions, we can confirm that eight variables, including “road density” and “population density,” are key factors influencing “folk building density.” These variables demonstrated significant correlations within the model and exhibited high reliability across different data subsets. The coefficients obtained from both regression methods for the key variables were relatively consistent, indicating that Elastic Net regression provided a more balanced approach, offering stable and precise coefficient estimates while effectively selecting relevant variables.

Additionally, due to regional differences, the impact of environmental, social, and economic activities varies, potentially leading to different effects on the clustering of Bengke buildings across various areas. We first divided the dataset into high-density and low-density groups based on building density differences across villages and towns to verify this. We then conducted regional regression analysis using the key factors identified in the earlier analysis to compare the influence of these factors across different regions [60, 61].

**Fig 11:**
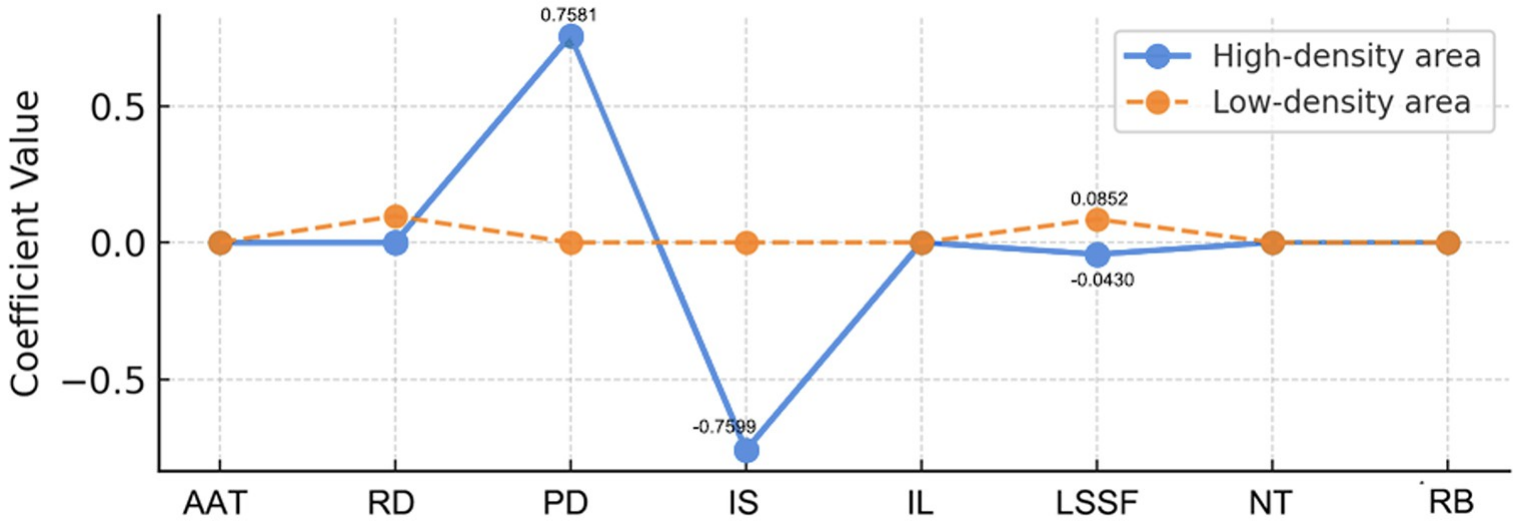
Comparison of coefficients in high-density and low-density area (self-illustration).

Given the small sample size, we continued using Lasso regression for this analysis (see Table 2 and Fig 11). Lasso regression is particularly effective in preventing overfitting in small sample cases and in identifying the most important variables [62], as follows:

**Table 2:** Factors influencing high and low aggregation areas.

Main influence factors	High-density area	Low-density area
**Average annual temperature**	0.0000	0.0000
**Road density**	0.0000	0.0965
**Population density (persons/km^2^)**	0.7581	0.0000
**Industrial structure**	−0.7599	0.0000
**Infrastructure Leve**	0.0000	0.0000
**Level of social service facilities**	−0.0430	0.0852
**Number of temples**	0.0000	0.0000
**Religious belief**	0.0000	0.0000

The analysis of the figures and tables reveals that in high-density areas, the concentration of traditional residences is significantly influenced by population density (coefficient of 0.7581) and industrial structure (coefficient of −0.7599). An increase in population density is often associated with the concentrated preservation and reuse of these traditional residences.

Conversely, an economy based on traditional agriculture and rural tourism may limit the expansion of new buildings, thereby contributing to the preservation of these century-old residences. In low-density areas, development is more dependent on infrastructure improvements. For instance, the enhancement of road density (coefficient of 0.0965) and the level of social service facilities (coefficient of 0.0852) not only fosters concentrated building development but also creates better conditions for the protection and utilization of traditional residences.

Therefore, high-density areas should focus on the preservation and reasonable utilization of traditional residences, while low-density areas should prioritize improving infrastructure quality to support the ongoing preservation and development of these buildings. This approach ensures that both the conservation of cultural heritage and the sustainable development of the region are effectively addressed.

## Discussion

This study systematically analyzed the spatial distribution and influencing factors of the Tibetan residential architecture “Bengke” in Luhuo County, a region renowned for its rich cultural heritage. Through the application of spatial statistical methods, we discovered that Bengke buildings exhibit significant clustering in certain areas, primarily influenced by environmental and socio-economic factors [63]. The findings indicate that elevation gradients, resource availability, and proximity to socio-economic activities play crucial roles in the spatial organization of these traditional buildings.

By employing a comprehensive analytical approach from multiple perspectives, this study identified key influencing factors. Specifically, the combined use of spatial statistical methods provided a thorough assessment of the spatial characteristics of Bengke buildings, while Lasso and Elastic Net regression methods allowed for the precise extraction of the most impactful variables, such as road density, population density, and economic development quality [45, 64]. These factors not only affect the spatial distribution of the buildings but also offer deep insights into broader socio-economic and environmental dynamics [28]. Additionally, this study explored the varying impact of these key factors in high-density and low-density areas, enhancing our understanding of how these factors interact and influence the distribution differences of traditional buildings [61, 65]. In high-density areas, population density and industrial structure are dominant factors, whereas in low-density areas, infrastructure quality and the level of social services have a more significant impact [26]. This differentiation highlights the importance of tailored strategies for the protection and development of Bengke buildings.

The results of this study hold significant implications for the preservation and sustainable development of Bengke architecture. By understanding the key driving factors behind spatial patterns, policymakers and conservationists can develop targeted strategies to protect this cultural heritage [66]. Furthermore, this study provides a methodological framework that can be applied to other regions with similar cultural and architectural significance, guiding related conservation efforts [67].

Despite the valuable insights provided by this study, there are some limitations. Future research could benefit from expanding the sample size, creating a more comprehensive and detailed dataset, and conducting cross-regional horizontal or longitudinal comparisons to gain a deeper understanding of the spatiotemporal distribution and dynamic changes of traditional buildings [68]. Additionally, exploring the impact of urbanization on the preservation of traditional buildings from the perspectives of urban development and cultural industry structure is a worthwhile direction for further investigation [69].

## Conclusion

This study through the integrated use of spatial statistical analysis and regression models, has uncovered the spatial distribution characteristics of Bengke architecture in Luhuo County, Western Sichuan, and identified the primary factors influencing this distribution. The findings indicate that the distribution of these buildings is not random but is shaped by a combination of environmental and socio-economic factors, with road density, population density, and economic development quality playing particularly significant roles. Additionally, the key influencing factors vary notably across different regions, suggesting that regional characteristics should be carefully considered when developing strategies for the protection and development of these structures.

While this research highlights the complexity and diversity of the spatial distribution of Bengke buildings, further studies are needed to expand the sample size and incorporate long-term dynamic data. Such research could provide deeper insights into the pathways for preserving and transmitting these traditional buildings amid ongoing social and economic changes.

## Supporting information

S1 File Sample village coordinates. (ZIP)
